# Increased basal insulin sensitivity in late pregnancy in women carrying a male fetus: a cohort study

**DOI:** 10.1186/s13293-022-00429-z

**Published:** 2022-05-04

**Authors:** Clive J. Petry, Ieuan A. Hughes, Ken K. Ong

**Affiliations:** 1 Department of Paediatrics, University of Cambridge, Cambridge Biomedical Campus, Hills Road, Box 116, Cambridge, CB2 0QQ UK; 2 MRC Epidemiology Unit, University of Cambridge, Cambridge, UK; 3 Institute of Metabolic Science, University of Cambridge, Cambridge, UK

**Keywords:** Development, Fetal sex, Gestational diabetes, Insulin secretion, Glucose

## Abstract

**Background:**

It has been suggested that fetal sex may be able to modify maternal metabolism and physiology during pregnancy. Recently pregnant women carrying a male fetus were reported to be more insulin sensitive than those carrying females, although related evidence is inconsistent.

**Methods:**

In this study we administered a 75 g oral glucose tolerance test at around week 28 of pregnancy in 813 pregnant women from a contemporary birth cohort (the Cambridge Baby Growth Study), derived surrogate indices of insulin secretion and sensitivity, and related them to the fetal sex.

**Results:**

Carrying a male fetus was associated with lower fasting glucose (difference in mean concentrations ≈ 0.1 mmol/L; *β*′ = 0.063; *p* = 0.02) and insulin (≈ 1.1 pmol/L; *β*′ = 0.075; *p* = 0.01) concentrations but not with post-load glucose or insulin concentrations. Male fetal sex was also associated with lower HOMA IR (≈ 1.08 units; *β*′ = 0.071; *p* = 0.02) and higher QUICKI (≈ 1.06 units; *β*′ = 0.080; *p* = 0.007) values suggesting increased basal insulin sensitivity. There were no differences in indices of insulin secretion, except for the insulin disposition index which was higher in women carrying a male fetus (≈ 1.15 units; *β*′ = 0.090; *p* = 0.007). Birth weights were higher in male offspring.

**Conclusions:**

Women carrying a male fetus were relatively more insulin sensitive in the fasting state and secreted more insulin relative to this degree of insulin sensitivity. These results are consistent with the idea that the fetal sex may be able to modify the maternal glucose-insulin axis.

## Highlights

- Pregnant women carrying a male fetus were more insulin sensitive than those carrying a female at around week 28 of pregnancy in a contemporary birth cohort.
- They also appeared to secrete more insulin relative to their degree of insulin sensitivity.
- The differences in glucose and insulin concentrations were evident in the basal state but disappeared after the consumption of a glucose load.
- Fetal sex appears to be able to influence maternal glucose and insulin concentrations in pregnancy.

## Background

Pregnancy represents an almost unique phase of human life where circulatory systems associated with two different individuals are able to directly interact with each other; the only other scenario when this can occur is in conjoined twins. In pregnancy the interaction between circulatory systems primarily involves the mother’s metabolism supplying the fetus with oxygen and nutrients via the placenta once it has developed, and then the safe removal of toxins and waste products from the fetal circulatory system. While the fetus is totally dependent upon the maternal metabolism for these actions, there is evidence that the fetus can modify the maternal metabolism and physiology [1].

One way that the fetus appears to be able to modify the maternal metabolism and physiology is via imprinted genes, which are expressed in the fetus in a parent-of-origin specific manner (one copy of the gene not being expressed, which one depending upon the gene and tissue in question, and the stage of development). Many of these genes are only expressed in utero [2] and they appear to be able to regulate placental endocrine function [3]. Thus, we have found associations between polymorphic variation in parental-specific fetal imprinted gene alleles and maternal glucose [4, 5], lipid species [6] and circulating DLK1 protein [7] concentrations in pregnancy. Similar results regarding glucose concentrations were also found using a knockout mouse model [8]. We have also found associations between various fetal imprinted gene alleles at polymorphic loci and maternal blood pressure in pregnancy [9]. We suggested that the mechanism underpinning the associations between parental-specific fetal imprinted gene alleles and changes in maternal metabolism and physiology may be via the alteration of placental hormone concentrations secreted into the maternal circulation [1], especially since much of the placenta is fetal in origin [10].

Another way that the fetus might be able to modify maternal metabolism and physiology (again possibly via alterations in placental endocrine function [11]) relates to the sex of the fetus. As examples of this, systematic reviews and meta-analyses of individual studies, as well as certain individual studies, have shown that mothers carrying male fetuses are more likely to develop gestational diabetes (GDM) than those carrying female fetuses [12–14]. However, in one recent study, independent of GDM risk, markers of insulin resistance were lower in pregnant women carrying male fetuses [15]. An earlier study failed to find a fetal sex effect on maternal insulin sensitivity [16], instead finding mothers carrying male fetuses having higher circulating glucose concentrations but lower pancreatic *β*-cell function. Another study found that mothers carrying male fetuses were actually more insulin resistant than those carrying females, when tested early in pregnancy [17]. A recent review of how placental endocrine function varies according to fetal sex called for more studies of fetal sex-mediated differences in the regulation of glucose metabolism in pregnancy in relatively homogeneous populations to try and clarify discrepancies in the literature in this area [18]. We therefore sought clarification of this matter using a largely ethnically homogeneous contemporary birth cohort, the Cambridge Baby Growth Study (CBGS).

## Methods

### Cambridge baby growth study

Between the years 2001 and 2009 early pregnancy ultrasound clinics held at the Rosie Maternity Hospital, Cambridge, U.K. (a single centre) were used to recruit 2229 pregnant women (and subsequently their babies and partners) to the observational CBGS [19]. All the women were over 16 years of age and were able to give consent for themselves and their baby. The CBGS was run prospectively and longitudinally. A total of 1658 of the women still wanted to be part of this study at the birth of their baby (571 women withdrew prior to this). A vast majority (95.3%) of the babies were ethnically White. Other babies were Black (African or Caribbean) (1.3%), Asian (1.7%) or mixed race (1.7%).

### Oral glucose tolerance tests

Oral glucose tolerance tests (OGTTs) were performed in 1071 of the CBGS participants at a median (interquartile range) of 28.4 (28.1–28.7) weeks of gestation [4]. The 75 g glucose load was administered orally after an overnight fast and collection of venous blood samples for the measurement of circulating glucose and insulin concentrations. Further blood samples were collected for additional glucose measurements one hour after the administration of the glucose. Capillary glucose measurements were made using an Abbott Freestyle Mini (Abbott Diagnostics, Maidenhead, U.K.) 0, 30, 60, 90 and 120 min. after the administration of glucose [4] and the 120-min. glucose concentrations were used in this analysis. From March 2007 onwards a second set of venous samples was collected two hours after the glucose load. GDM in this study was classified from these venous (OGTT fasting and 60 min.) and capillary (OGTT 120 min.) glucose concentrations using International Association of Diabetes and Pregnancy Study Group criteria [20]. Biomarkers of fasting insulin resistance (HOMA IR, QUICKI, reciprocal fasting insulin concentration), pancreatic *β*-cell function (HOMA B) and insulin secretion (insulin increment, insulinogenic index and insulin disposition index) were calculated.

### Offspring size at birth

Birth weight was recorded from the hospital notes. Other measurements in newborns, such as the length, head circumference and various skinfolds thicknesses, were made as soon as possible after birth [at a median (interquartile range) age of 2 (1–16) days of age] by trained paediatric research nurses. Each measurement was performed in triplicate and the mean of these measurements used for statistical analyses. Body length was measured using a SECA 416 Infantometer (Hamburg, Germany). Skinfolds thicknesses at the subscapular, flank, triceps and quadriceps regions of the left side of the body were measured using Holtain calipers and head circumferences were measured using a tape measure (both Chasmors Ltd., London, U.K.) [21]. Intra- and inter-observer technical errors of measurement were 0.4–2.8% and 2.0–3.2%, respectively, for the skinfold thicknesses.

### Laboratory measurements

OGTT plasma insulin concentrations were measured using a Dako enzyme-linked immunosorbent assay (Dako UK Ltd., Ely, Cambs, UK). Intra-assay imprecision (%CV) values were 4.3% at 82 pmol/L, 3.0% at 402 pmol/L and 5.7% at 907 pmol/. Equivalent inter-assay imprecision values were 4.3, 5.1 and 5.4%, respectively. Venous blood glucose measurements were made using a routine glucose oxidase-based method (Yellow Springs International Inc., Yellow Springs, OH, USA).

### Calculations

The index of multiple deprivation was derived and imputed from the postcode of the participants’ home addresses as described [22]. HOMA IR and B were calculated using the fasting glucose and insulin concentrations and the online HOMA calculator [23]. QUICKI was also calculated using the fasting glucose and insulin concentrations, and the following equation (using insulin and glucose concentrations expressed in SI units): 1/(((log (insulin/6)) + (log (glucose*18))) [24]. The insulin increment was calculated as the OGTT fasting insulin concentration subtracted from the OGTT 60-min. insulin concentration. The insulinogenic index was calculated as the insulin increment divided by the rise in glucose concentrations over the first hour of the OGTT. The insulin disposition index was calculated as the insulinogenic index divided by the fasting insulin concentration. Dividing the maternal (pre-pregnancy) or baby weights by their height squared was used to calculate the body mass indexes (BMI).

### Statistical analysis

This analysis was restricted to those women who underwent OGTTs and for whom data relating to the gestational age of the baby when the OGTT was performed, pre-pregnancy BMI and maternal age were available (*n* = 813). Continuous variables were analysed in statistical models using linear and/or quadratic regression models, adjusted for confounders where appropriate. Where the dependent variable residuals were skewed, the models were analysed with prior (generally logarithmic) transformation of the data so that the residuals were normally distributed. Categorical variables were analysed using the *χ*^2^-test or Fisher’s exact test (as appropriate) or logistic regression. The risk ratio (RR) was calculated using Stata’s binreg function. *p* < 0.05 was considered statistically significant throughout. The statistical analyses were performed using either R (version 4.0.3; The R Foundation for Statistical Computing, Vienna, Austria) or Stata (version 13.1; Stata Corp., from Timberlake Consultants Ltd., Richmond, Surrey, U.K.). Data are mean (95% confidence interval) unless stated otherwise.

## Results

### Clinical characteristics of study participants

Those women who were included in this study largely shared similar clinical characteristics to those CBGS participants who were excluded from this study (Table 1), with the exception that there were more women who had not had a previous pregnancy, a lower proportion of these women smoked and they tended to put on more weight during pregnancy and gave birth around one day later. In those women who were included in this analysis, clinical characteristics were similar between those carrying a male fetus and those carrying a female fetus (Table 2).

**Table 1:** Clinical characteristics of the CBGS women included in this study and those excluded from it

Characteristic	Included	Excluded	*p*-value
Age (years)	33.5 (33.2, 33.8) (n = 813)	33.6 (33.2, 34.0) (n = 522)	0.6
Height (cm)	166.0 (165.5, 166.5) (n = 767)	165.7 (165.1, 166.4) (n = 484)	0.5
Pre-pregnancy weight (kg)	66.4 (65.4, 67.4) (n = 753)	66.1 (64.8, 67.3) (n = 464)	0.7
Pre-pregnancy BMI (kg/m^2^)	24.2 (23.9, 24.5) (n = 813)	24.1 (23.8, 24.5) (n = 624)	0.8
Weight gain in pregnancy (kg)	9.0 (8.4, 9.5) (n = 554)	6.9 (6.2, 7.7) (n = 320)	1.2 × 10^–5^
Parity (*n* 0/1/2/3/4/5)	405/289/89/22/4/3	312/351/126/39/4/3	2.5 × 10^–5^
Index of multiple deprivation	8.9 (8.6, 9.3) (n = 545)	9.0 (8.5, 9.4) (n = 268)	1.0
Smoked during pregnancy (yes/no)	28/785	58/784	2.0 × 10^–3^
Anaemia during pregnancy (*n* yes/no)	28/713	12/488	0.2
Length of pregnancy (weeks)	39.9 (39.7, 40.0) (n = 813)	39.7 (39.6, 39.8) (n = 844)	0.02

Data are mean (95% confidence interval) or number of participants

**Table 2:** Clinical characteristics of the study participants according to the fetal sex

Characteristic	Male fetus	Female fetus	*p*-value
Age (years)	33.5 (33.1, 33.9) (*n* = 422)	33.4 (33.0, 33.9) (*n* = 391)	0.9
Height (cm)	165.6 (165.0, 166.3) (*n* = 402)	166.4 (165.7, 167.2) (*n* = 365)	0.1
Pre-pregnancy weight (kg)	66.4 (65.1, 67.7) (*n* = 395)	66.4 (65.1, 67.8) (*n* = 358)	1.0
Pre-pregnancy BMI (kg/m^2^)	24.2 (23.8, 24.6) (*n* = 422)	24.2 (23.7, 24.6) (*n* = 391)	0.9
Weight gain in pregnancy (kg)	9.2 (8.4, 10.0) (*n* = 292)	8.8 (7.9, 9.6) (*n* = 262)	0.5
Parity (*n* 0/1/2/3/4/5)	204/159/45/9/1/3	201/130/44/13/3/0	0.3
Index of multiple deprivation	9.1 (8.6, 9.6) (*n* = 277)	8.8 (8.3, 9.3) (*n* = 268)	0.8
Smoked during pregnancy (yes/no)	15/407	13/378	0.9
Anaemia during pregnancy (*n* yes/no)	14/369	14/344	0.9
Length of pregnancy (weeks)	39.9 (39.7, 40.0) (*n* = 422)	39.9 (39.7, 40.0) (*n* = 391)	0.7

Data are mean (95% confidence interval) or number of participants

### Assessment of the glucose-insulin axis during pregnancy by fetal sex

**Fig. 1:**
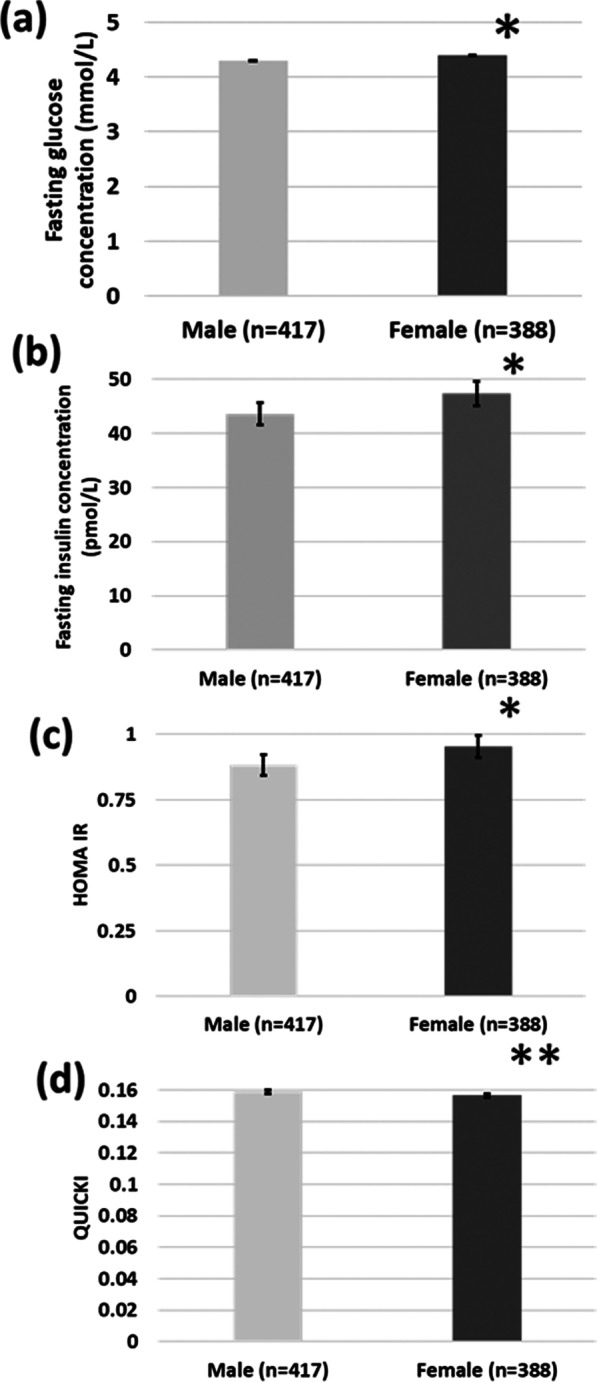
Fasting **a** glucose and **b** insulin concentrations, **c** HOMA IR values and **d** QUICKI values by fetal sex (all adjusted for pre-pregnancy BMI, maternal age, gestational age of the fetus and multi-fetal pregnancy). Data are mean ± 95% confidence interval. *p < 0.05, **p < 0.01

**Fig. 2:**
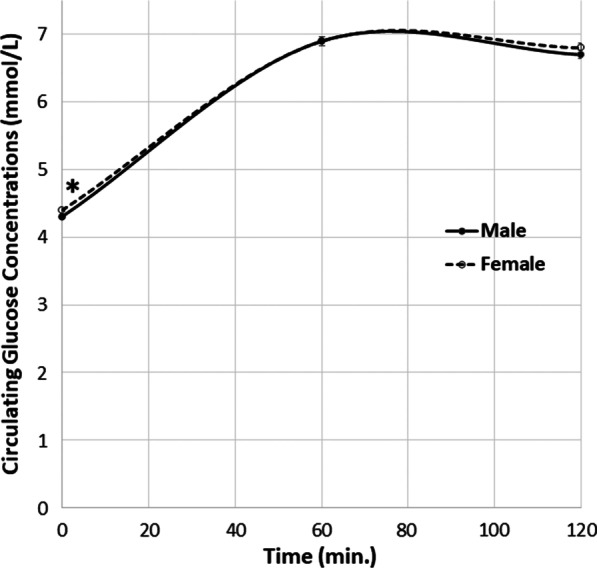
Circulating glucose concentrations (adjusted for pre-pregnancy BMI, maternal age, gestational age of fetus and multi-fetal pregnancy) in 75 g OGTTs by fetal sex. Data are mean ± SEM. *p < 0.05

OGTT fasting glucose concentrations were lower in women carrying male babies than in those carrying females (Fig. 1). There were no differences in circulating 60- or 120-min. glucose concentrations between those carrying male and female fetuses (Fig. 2). Surrogate markers of insulin resistance (higher HOMA IR, lower QUICKI, higher fasting insulin concentrations) were lower in women carrying male fetuses than in those carrying females (Fig. 1). Some of the surrogate markers of insulin secretion (insulin increment, insulinogenic index) showed no association with the fetal sex (Table 3). Neither was there a difference in the surrogate marker of pancreatic *β*-cell function (HOMA B). However, the insulin disposition index was higher in those women carrying males. There was no association between the fetal sex and the risk of GDM [RR (male to female) 1.4 (0.9–2.0); *n* = 813; *p* = 0.2].

**Table 3:** Selected OGTT assessment of the glucose-insulin axis, including surrogate indices of insulin secretion

Variable	*n*	Unadjusted		Adjusted*	
		*β*′	*p*	*β*′	*p*
HOMA B	813	− 0.035 (− 0.103, 0.034)	0.3	− 0.033 (− 0.095, 0.030)	0.3
OGTT 60-min. insulin concentration	805	0.036 (− 0.032, 0.104)	0.3	0.022 (− 0.040, 0.083)	0.5
OGTT 60-min. glucose concentration	806	0.014 (− 0.042, 0.071)	0.6	− 0.008 (− 0.057, 0.040)	0.7
OGTT 120-min. capillary glucose concentration	610	− 0.037 (− 0.118, 0.043)	0.4	− 0.052 (− 0.125, 0.020)	0.2
0–60-min. insulin increment	805	0.038 (− 0.029, 0.106)	0.3	0.026 (− 0.037, 0.088)	0.4
0–60-min. insulinogenic index	768	0.031 (− 0.037, 0.099)	0.4	0.040 (− 0.027, 0.108)	0.2
0–60-min. insulin disposition index	768	0.066 (− 0.005, 0.136)	0.07	0.090 (0.025, 0.156)	0.007

Where significant associations have positive standardised *β*′s, values from women carrying male fetuses are higher than those from women carrying females. Where significant associations have negative standardised *β*′s, values from women carrying female fetuses are higher than those from women carrying males

*Adjusted for pre-pregnancy BMI, maternal age, gestational age of fetus, multi-fetal pregnancy and GDM

### Offspring birth characteristics by fetal sex

As expected, male offspring were heavier, longer and had bigger head circumferences at birth (Table 4). Visual inspection of the data suggested that there were both linear and quadratic elements in both male and female offspring to the relationship between maternal fasting glucose concentrations and offspring birth weights. In interactive models (adjusted for maternal BMI, age, gestational age at OGTT and at birth, parity and multi-fetal pregnancies) the linear relationship was evident in both male (*β*′ = 2.013; *p* = 9.4 × 10^–4^) and female (*β*′ = 1.614; *p* = 6.8 × 10^–4^) offspring. Similarly, the quadratic relationship was also evident in both male (*β*′ = -0.763; *p* = 0.02) and female (*β*′ = − 0.517; *p* = 4.7 × 10^–3^) offspring. There was no difference in offspring BMI or ponderal index by sex of the offspring. However, skinfold thicknesses of the flank, quadriceps and subscapular regions were lower in boys than in girls. There were no differences in triceps skinfold thicknesses by sex.

**Table 4:** Offspring birth characteristics according to fetal sex

Variable	N	Unadjusted		Adjusted*	
		*β*′	*p*	*β*′	*p*
Weight	810	0.112 (0.046, 0.178)	9.0 × 10^–4^	0.120 (0.065, 0.174)	2.1 × 10^–5^
Length**	786	0.164 (0.098, 0.231)	1.4 × 10^–6^	0.161 (0.113, 0.210)	1.5 × 10^–10^
Head Circumference**	786	0.191 (0.124, 0.257)	2.8 × 10^–8^	0.187 (0.137, 0.237)	4.5 × 10^–13^
Body mass index**	784	0.008 (− 0.061, 0.077)	0.8	0.016 (− 0.038, 0.071)	0.6
Ponderal index**	784	− 0.058 (− 0.127, 0.012)	0.1	− 0.048 (− 0.101, 0.005)	0.07
Flank skinfolds thickness**	787	− 0.094 (− 0.163, − 0.026)	6.8 × 10^–3^	− 0.097 (− 0.160, − 0.035)	2.4 × 10^–3^
Quadriceps skinfolds thickness**	786	− 0.105 (− 0.171, − 0.039)	2.0 × 10^–3^	− 0.107 (− 0.163, − 0.052)	1.5 × 10^–4^
Subscapular skinfolds thickness**	788	− 0.085 (− 0.151, − 0.018)	0.01	− 0.089 (− 0.149, − 0.028)	4.2 × 10^–3^
Triceps skinfolds thickness**	785	− 0.028 (− 0.096, 0.039)	0.4	− 0.032 (− 0.095, 0.031)	0.3

Males larger than females shown as positive associations, and females larger than males shown as negative associations

*Adjusted for pre-pregnancy BMI, gestational age of fetus, multi-fetal pregnancy and maternal parity

**Values additionally adjusted (in the pre-adjusted models) for age at time of measurement

## Discussion

In this study we have found pregnant women carrying male fetuses had lower fasting circulating glucose and insulin concentrations than those carrying females at around week 28 of pregnancy. Surrogate indices also suggest that those carrying male fetuses were more insulin sensitive, which is consistent with some [15, 25] but not all [16, 17] published studies. We also found that women carrying male fetuses had higher insulin disposition indices, suggesting higher insulin secretion for their degree of insulin sensitivity. We hypothesise that higher insulin sensitivity and lower glucose concentrations may reduce growth stimulation in male fetuses, given the observed relationship between maternal fasting glucose concentrations and offspring birth weights. This could enhance survival, with male fetuses being more prone to high birth weights [26] and such babies being at increased risk of neonatal death [27].

Despite the differences in fasting concentrations, we observed no associations between the fetal sex and OGTT post-load glucose and insulin concentrations, so there was no evidence of altered non-basal insulin sensitivity. The reduced insulin resistance and circulating glucose concentrations observed in the fasting state of pregnant women carrying male fetuses may therefore relate more to reduced hepatic glucose output than to increased insulin-stimulated glucose uptake into muscle or adipose tissue [28] as hepatic glucose output is thought to have a greater influence on fasting than post-prandial glucose concentrations. One plausible mechanism of this involves differences in placental structure or function, most of the placenta being fetal in origin [10, 18], leading to differential secretion of placental hormones and/or other biologically active molecules into the maternal circulation. Of the various placental hormones whose maternal circulating concentrations differ according to fetal sex [18], one candidate which has been shown to be secreted in decreased amounts into the circulation with male fetuses [11] is human placental lactogen (hPL). hPL stimulates increased circulating non-esterified fatty acid levels [29], which in turn can increase hepatic glucose output [30] and glucose-stimulated insulin secretion [31]. All these lead to hPL inhibiting insulin sensitivity [32]. However, it also increases β-cell mass and function [18], which is inconsistent with the present results. Testosterone, a candidate hormone secreted into the circulation in increased amounts in women carrying male fetuses [33] (although not universally [34]), is unlikely to mediate the observed associations since it has been associated with insulin resistance [35, 36]. Another potential candidate hormone is oestradiol, whose circulating concentrations are lower in women carrying males, at least in early pregnancy [37]. These reduced oestradiol concentrations are associated with increased insulin sensitivity [18]. Like the role of hPL, therefore, the role of oestradiol in affecting insulin sensitivity and secretion in pregnancy is only partially consistent with what was observed in the present analysis. Non-hormonal candidate molecules that may alter insulin sensitivity in pregnancy [38] and show fetal sex-related differences [39] include regulatory cytokines, although these are less well studied in terms of effects on the glucose-insulin axis in pregnancy.

In the present study there was no observable significant association between fetal sex and risk of GDM, which is inconsistent with meta-analyses of the subject [12, 13]. However, the direction and magnitude of the non-significant association in the present study was consistent with these studies. Also, closer inspection of those original studies included in the meta-analyses show that 15 out of 21 exhibited no significant risk according to fetal sex in one publication [12] and 21 out of 28 in another [13].

As expected, in the present study there were differences in size at birth by offspring sex. Males had higher birth weights, lengths and head circumferences. Females had regions of greater adiposity, as suggested by increased skinfold thicknesses in the flank, quadriceps and subscapular regions, as observed previously [17, 40]. This decreased adiposity in males was regionalised as it was not observed with the triceps region. Neither were there any differences in overall adiposity in terms of the ponderal index and BMI. The decreased adiposity observed in the three other skinfolds regions may relate to the increased insulin sensitivity in women carrying males, as it has previously been observed to relate to the offspring fat mass [41].

The strengths of this study include the use of a relatively large contemporary birth cohort with detailed measurements relating to the maternal glucose-insulin axis and offspring birth characteristics. Its limitations include possible biases introduced by only studying a proportion of the full cohort. However, there was no apparent (fetal) sex bias in clinical characteristics that showed differences between those included and excluded from this study. Another limitation is that the cohort itself may not be representative of the general population. However, the demographics of the cohort are very similar to those of pregnancies of the Rosie Maternity Hospital overall [19]. A third limitation is the lack of OGTT 30-min. insulin concentrations, which are usually used for the calculation of indices of insulin secretion. Nevertheless, in previous OGTT studies the 60-min. insulins correlate well with the 30-min. values [42] suggesting that our calculated values should still be useful surrogates.

## Conclusions

In conclusion our results from this study are consistent with the idea that the fetal sex may be able to influence the maternal glucose-insulin axis, with pregnant women carrying males having slightly lower glucose concentrations and being more insulin sensitive in the basal state than those carrying females. Our results suggest that this may only be evident when fasted, although further work is needed to clarify this.

## Perspectives and significance

Results from the present analysis add to those in the literature suggesting that in pregnancy the maternal glucose-insulin axis can be influenced by the fetal sex, albeit our associations had modest effect sizes. Unfortunately, these actual potential effects of fetal sex appear to vary according to the makeup of the populations tested [12–18]. Our results come from a relatively homogeneous cohort living in a comparatively affluent area of England [19], potentially limiting confounding. Further analyses could usefully delineate what mediates the heterogeneity of results from different studies and which placental hormones and/or bioactive molecules mediate the associations.

## Data Availability

The dataset analysed during the current study is available in the Apollo repository, https://doi.org/10.17863/CAM.58801.
